# Structural Elucidation of a Polysaccharide from *Flammulina velutipes* and Its Lipid-Lowering and Immunomodulation Activities

**DOI:** 10.3390/polym16050598

**Published:** 2024-02-22

**Authors:** Wei Jia, Wenhan Wang, Dongsheng Yu, Yangchao Yu, Zhan Feng, Hewen Li, Jingsong Zhang, Henan Zhang

**Affiliations:** 1 Institute of Edible Fungi, Shanghai Academy of Agricultural Sciences, Key Laboratory of Edible Fungal Resources and Utilization (South), Ministry of Agriculture and Rural Affairs, P. R. China, National Engineering Research Center of Edible Fungi, National R&D Center for Edible Fungal Processing, Key Laboratory of Agricultural Genetics and Breeding of Shanghai, Shanghai 201403, China; jiawei@saas.sh.cn (W.J.); wangwenhan@saas.sh.cn (W.W.); dongsheng.yu@jwtherapeutics.com (D.Y.); 2 Jiangsu Chinagreen Biotechnology Co., Ltd., Suqian 223700, China

**Keywords:** *Flammulina velutipes*, polysaccharides, three-phase partitioning, structural characteristics, lipid-lowering activity, immunoregulatory activity

## Abstract

FVPT1, a novel heteropolysaccharide, was purified from the fruiting body of *Flammulina velutipes* using magnetic-field-assisted three-phase partitioning and gel permeation chromatography. The structure was characterized using monosaccharide composition and methylation analysis, infrared spectroscopy and nuclear magnetic resonance (NMR). The FVPT1 (~1.64 × 10^4^ Da) was composed of L-fucose, D-galactose, D-glucose and D-mannose at a molar ratio of 1.0:3.5:1.0:1.4. The polysaccharide repeating unit of FVPT1 was established with methylation analyses and NMR spectroscopy. Moreover, a zebrafish larva hyperlipidemia model test demonstrated that FVPT1 can show appreciable lipid-lowering effects. In addition, the FVPT1 exhibited remarkable immunoregulatory activity by increasing nitric oxide, interleukin (IL)-1β and IL-1 secretion in macrophages. Therefore, these results suggest that FVPT1 has the potential to be developed into a new immune or hypolipidemic health product.

## 1. Introduction

*Flammulina velutipes*, also known as the golden needle mushroom, ranks fourth in the world in terms of the production and consumption of edible mushrooms [1]. *F. velutipes* is rich in carbohydrates, protein and vitamins, which makes it popular among consumers in Asia, especially China and Japan. The polysaccharides, glycoproteins, phenols and sesquiterpenes isolated from *F. velutipes* have multiple pharmacological activities, such as antitumor, anti-inflammatory, antioxidant, hypolipidemic, immunity-regulation and other health-promotion effects [2,3,4,5,6,7,8]. *F. velutipes*’ polysaccharides, as some of its most important active components, have been studied for many years [9,10]. The polysaccharides extracted from *F. velutipes* using different methods have had remarkable therapeutic effects and undetected toxicity, which has attracted widespread attention [11,12]. Many studies have shown that different extraction methods can affect extraction yield, structural characteristics and biological activity. As described by Guo et al. [13], microwave-assisted extraction of polysaccharides has more obvious antioxidant activity than do hot-water and ultrasound-assisted extraction. Another study showed that the microwave extraction of *Cordyceps gunnii* mycelia polysaccharides demonstrated the highest yield and antitumor activity, as well as the largest macromolecular polysaccharide ratio [14]. At present, a variety of extraction methods of *F. velutipes* polysaccharides, mainly including hot-water and microwave-assisted, ultrasonic-assisted and enzyme-assisted extraction, have been widely used in research. For example, Zhang et al. [15] found that the maximum extraction yield of *F. velutipes* polysaccharides arrived at 8.33% with 620 W for 20 min at 45 °C. Although traditional extraction methods are relatively simple, their shortcomings, such as time-consuming treatment and complicated separation and purification steps, subsequently cannot be ignored. As a new method of polysaccharide extraction, three-phase partitioning (TPP) has become more and more widely used due to its high efficiency and semipurification characteristics.

As a normal method of separation and purification of proteins and oils, three-phase partitioning technology (TPP) is a new, safe and green technology including salting out, isoelectric point precipitation and solvent precipitation using t-butanol and ammonium sulfate solution successively to obtain the upper organic phase, the intermediate protein phase of the interfacial precipitate and the lower aqueous phase [16,17,18]. Currently, TPP has also been widely applied as a more efficient and fast extraction technology to extract polysaccharides such as those of *Corbicula fluminea*, xylanase isolated from *Bacillus oceanisediminis* strain SJ3 and those of aloe [19,20,21]. In addition, the magnetic-field-enhanced extraction method called “green separation technology” uses its field to enhance the chemical separation process. It can use the special energy generated by the magnetic field to change the properties of diamagnetic substances by changing their microstructure. In a research paper about magnetic-field-assisted solvent extraction, published by Palyska [22], D-2EHPA (di-2-ethylhexyl phosphoric acid) was used to extract Cu^2+^, and the distribution ratio of copper increased 160 times after the magnetization of the extractant. Another article found that a variable magnetic-field-assisted extraction technique made it possible to extract more mineral components, caffeine and polyphenols from dry black and green tea at a frequency of 50 Hz compared to conventional extraction methods [23]. Therefore, magnetic-field-assisted three-phase partitioning was used to separate *F. velutipes* polysaccharides in this study in hopes of obtaining new *F. velutipes* polysaccharides with higher yields and purity.

In this study, the structure of *F. velutipes* polysaccharide FVPTI, extracted using magnetic-field-assisted TPP and gel permeation chromatography, was analyzed using monosaccharide composition and methylation analyses, as well as NMR. In addition, the lipid-lowering and immunological activities of FVPT1 are also discussed. This study will provide a theoretical basis for the future large-scale industrial production of this polysaccharide as well as active polysaccharide materials for the pharmaceutical, the food and other industries.

## 2. Materials and Methods

### 2.1. Materials and Chemicals

Fresh *F. velutipes* fruiting bodies were collected from Jiangsu Chinagreen Biotechnology Co., Ltd. (Suqian, China). Standard monosaccharides were purchased from Sigma-Aldrich Chemical Co. (St. Louis, MO, USA). Zebrafish larva AB strains (Nanjing YSY Biotech Company Ltd. (Nanjing, China)), egg yolk powder (Shangdong Xitang Company (Jinan, China)), and an ELISA kit for cytokine assays (Becton, Dickinson and Company (New York, NY, USA)) were also used.

### 2.2. Isolation and Purification of F. velutipes Polysaccharides Using Magnetic-Field-Assisted Three-Phase Partitioning

**Figure 1:**
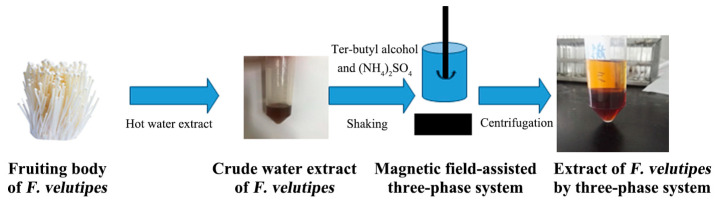
Extraction and separation steps for polysaccharides from *F. velutipes* using the three-phase partitioning (TPP) system.

The fruiting body of *F. velutipes* was washed, dried, crushed and sieved at 100 mesh. The powder of the *F. velutipes* was suspended in 500 mL of 95% ethanol and extracted for 1 h with an SB25-12D ultrasonic generator (Ningbo Scientz Biotechnology Co., Ltd. (Ningbo, China))at a 40 kHz frequency three times and hot water extraction three times, each time for 2 h. Certain amounts of solid ammonium sulfate (mass fraction 10–60%) and tert-butyl alcohol (5–30 mL) were added to the water extract at room temperature. The extraction tube was placed under a magnetic field, stirred at a certain rotation speed of 100 rpm for 30 min and then used to form three clear phases at 2500 rpm for 5 min. The lower aqueous phase was dialyzed, concentrated and dried to obtain purified extracted *F. velutipes* polysaccharides (Figure 1).

Further purification used gel permeation chromatography (GPC) (high-resolution Sephacryl S-300 column) (XK16 × 100 cm) (GE Healthcare (Cardiff, UK)), and filtered, distilled water was used as the eluent. Two peaks were detected using a refractive index detector (RID-10A, Shimadzu Corporation (Kyoto, Japan)), and the first peak was collected and designated as FVPT1.

### 2.3. Monosaccharide Composition Analysis

The sample (2 mg) was hydrolyzed completely at 110 °C using 2 M TFA (Trifluoroacetic acid) for 4 h. High-performance anion exchange chromatography (HPAEC) equipped with a CarboPac Dionex LC30 ™ PA20 column (3 mm × 150 mm) (Thermo Fisher Scientific Inc. (Waltham, MA, USA)), eluted with 2 mM of NaOH and 0.05–0.2 M NaAc (0.45 mL/min), was used to detect sugar composition with a pulse amperometric detector (Dionex), using monosaccharides as standards.

### 2.4. Determination of Purity and Molecular Weight

The purity and molecular weight of the sample was analyzed with high-performance liquid size exclusion chromatography (HPSEC) equipped with a refractive index detector (RI) and TSK PWXL 6000 and 3000 gel filtration columns; eluted with a PB buffer at 0.5 mL/min; and calibrated with pullulan standards of P5 (6200 Da), P10 (10,000 Da), P20 (21,700 Da), P100 (113,000 Da) and P200 (200,000 Da) (Shodex, Tokyo, Japan) at 35 °C.

### 2.5. Fourier-Transform Infrared (FTIR) Spectroscopy

The FVPT1 was ground with KBr powder and made into KBr discs after being mixed evenly for transformation infrared spectra analysis in a Perkin–Elmer 599B FTIR spectrophotometer (Waltham, MA, USA) in the wavenumber region of 4000–400 cm^−1^ at a 4 cm^−1^ resolution, with 32 sample scans [24].

### 2.6. Methylation Analysis

Methylation analysis of the FVPT1 (2 mg) was conducted according to a previous study [25]. The methylated polysaccharide was then converted into partially methylated alditol acetates (PMAAs) using hydrolysis, reduction with sodium borodeuteride and acetylation. Glycosidic linkage analysis was performed via a GC-MS system (Thermo Finnigan TRACE 2000/MS) (Thermo Fisher Scientific Inc. (Waltham, MA, USA)) equipped with a DB-5 MS column (30 m × 0.25 mm, 0.25 µm and 0.2 mm film thicknesses). The temperature was programmed from 180 to 270 °C at 20 °C/min and held at 270 °C for 25 min [26]. The individual peaks of the PMAA and fragmentation patterns were identified using their mass spectra and relative retention times in the NIST 2011 database of GC-MS. Percentages of methylated sugars were estimated as ratios of the peak areas.

### 2.7. Nuclear Magnetic Resonance (NMR) Analysis

The FVPT1 was dissolved with D_2_O and lyophilized in a vacuum freeze dryer to facilitate deuterium exchange. The deuterium-exchanged FVPT1 (40 mg) was dissolved in 0.5 mL of 99.96% D_2_O for NMR. ^1^H,^13^C, Nuclear Overhauser Effect Spectroscopy (NOESY), heteronuclear multiple quantum coherence (HMQC) and ^1^H-detected heteronuclear multiple-bond correlation (HMBC) NMR spectra were recorded at 27 °C on a Bruker Avance III 600 MHz NMR spectrometer (Bruker Corporation (Billerica, MA, USA)). ^1^H chemical shifts were referenced to residual HDO, with δ 4.78 ppm (27 °C) as the internal standard. ^13^C chemical shifts were determined in relation to DSS (δ 0.00 ppm) calibrated externally. The ^1^H-^1^H-correlated spectroscopy (COSY) and HMQC were used to assign signals. HMBC and NOESY were used to assign inter-residue linkages and sequences.

### 2.8. Determination of NO from Macrophages

RAW264.7 mouse macrophages in the logarithmic growth phase were diluted into 2 × 10^5^ cells/well with the colorless medium DMEM, and the number of cells per well was as similar as possible. After 4 h in the incubator, samples were added to the cells, followed by observation of the adherence of the cells under the microscope and discarding of the supernatant. The 5 mg/mL *F. velutipes* polysaccharide solution was diluted to 100, 200 and 500 μg/mL with RPMI-1640. Then, 96-well plates were added to 200 μL of each well. The negative and positive control media, containing 5% PBSs and 10 μg/mL of LPSs (lipopolysaccharides), respectively, were set up with six replicates each. After 48 h of incubation at a constant temperature with a 5% carbon dioxide incubator atmosphere, 100 μL of supernatant was taken and detected with a Griess experiment to determine NO levels, as has been described previously [27].

### 2.9. Detection of Cytokine Secretion in Macrophages Using ELISA

IL-1 and IL-1β released by macrophages were detected with an ELISA kit. The macrophage suspension was diluted to 2 × 10^6^ cells/mL and cultured in a 96-well plate with an FVPT1 solution at a concentration of 200 μg/mL in each well. RPMI-1640 media containing 0.5% PBS and 10 μg/mL of LPS were used as negative and positive controls, respectively, followed by the setup of three replicates. The levels of IL-1 and IL-1β in the supernatant were determined using ELISA, with reference to the operation steps of the kit instructions after culturing for 4, 8 and 10 days at 37 °C and 5% CO_2_, respectively.

### 2.10. Lipid-Lowering Activity of FVPT1

#### 2.10.1. Zebrafish Larva Hyperlipidemia Model

The embryos of zebrafish of the AB strain were obtained from Nanjing YSY Biotech Company Ltd. (Nanjing, China) and incubated in clean fish water with oxygen for 48 h at 28 °C. The model of hyperlipidemia was established by giving yolk powder as a high-cholesterol diet (HCD) feed to the zebrafish larvae for six days [28]. The zebrafish larvae were divided into a control group, a high-cholesterol group and a sample group (100, 200 and 400 μg/mL of FVPT1, respectively) at a density of 10 zebrafish per group, placed in 12-well plates containing 2 mL of fish water per hole for treatment. In the high-cholesterol group, the yolk powder was used as HCD feed for the zebrafish larvae, with a final concentration of 0.1%, while the control group was not fed. From the 12th hour of feeding the zebrafish larvae with the high-cholesterol egg yolk diet, the lipids in the larvae began to accumulate rapidly. Soaking zebrafish larvae with egg yolk powder for 48 h can induce a large amount of lipid accumulation in the larvae and establish a hyperlipidemia model. In the high-cholesterol polysaccharide group, the polysaccharide and egg yolk powder were added into the fish water to soak the zebrafish larvae for 48 h. The lipid accumulation in the larvae was observed using oil red staining.

#### 2.10.2. Lipid Oil Red Staining in Zebrafish Larvae

An oil-red-O dye solution was configured according to instructions and slowly filtered with qualitative filter paper. The prepared solution was used up within 2 h. The zebrafish larvae were immobilized with polyformaldehyde (4%) for 1 h and then washed with PBS 2–3 times, followed by dehydration with methanol solvent in gradients of 25%, 50%, 75% and 100%, successively. After oil-red-O dyeing for 2 h, 100%, 75%, 50% and 25% gradient methanol solvent were used for gradient elution, followed by washing with PBS. Finally, fluorescence microscopy was used to observe and take photos.

#### 2.10.3. Intensity Quantification

After the oil-red-O staining, lipid accumulation could be clearly observed in the blood vessels and stomachs of the zebrafish larvae. Image-Pro Plus (IPP) analysis software (version 6.0) was used to determine the integrated optical density (IOD) value of the dyed lipids in the zebrafish, to quantitatively evaluate the level of lipid accumulation in vivo and then to analyze the inhibitory activity of the polysaccharide samples on the lipid accumulation in the larvae [29]. The normalized degree of lipid accumulation in vivo (%) = (IODpolysaccharide group − IODcontrol group)/(IODhyperlipidemia group − IODcontrol group).

### 2.11. Statistical Analysis

All experiments were performed in triplicate, and the data are expressed as means ± standard deviations (SDs). One-way analysis of variance (ANOVA) and the LSD test were used for intergroup comparisons. All variables were given Levene’s test for normality and homogeneous variance. If necessary, Tamhane’s T2 test was performed. *p* < 0.05 and *p* < 0.01 were significant and very significant, respectively.

## 3. Results and Discussion

### 3.1. Purification Results of FVPT1

The yield, polysaccharide content and protein content of the *F. velutipes* polysaccharide obtained using the magnetic-field-assisted three-phase partitioning were 2.3%, 55.53% and 12.19%, respectively. The polysaccharide content of the FVPT1 obtained after purification with gel-permeation chromatography reached 90.1%, and the moisture content was 2.91%.

### 3.2. Composition and Structural Characterization of FVPT1

**Figure 2:**
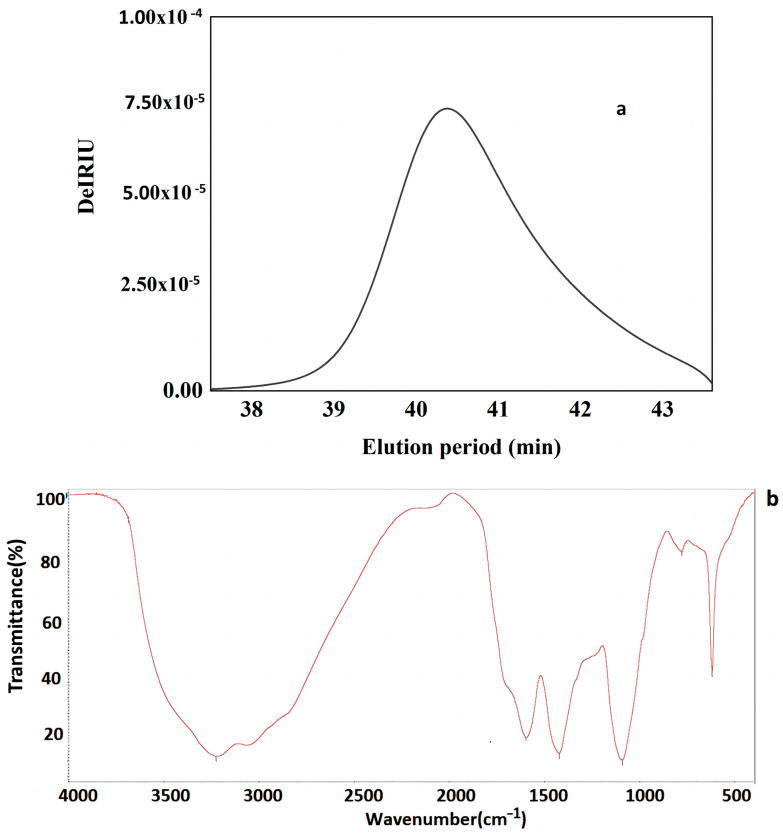

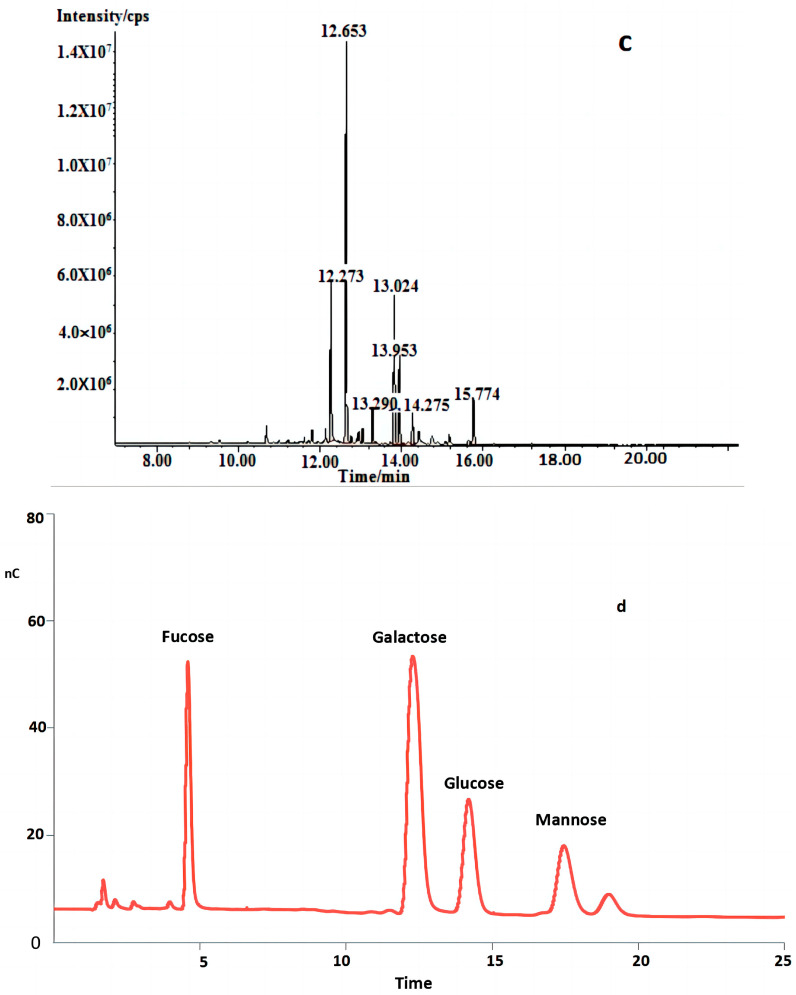
(**a**) HPLC profiles of FVPT1 (black line represents FVPT1). (**b**) FT-IR spectrum of FVPT1. (**c**) Total ion spectrum of FVPT1. (**d**) Spectrum of HPAEC of FVPT1.

The total polysaccharide content and protein content of the FVPT1 were 90.1% and 5.20%, respectively. A single symmetrical peak (Figure 2a) in the HPSEC profile indicated that FVPT1 is a homogeneous polysaccharide, with ~1.64 × 10^4^ Da and galactose (Gal), mannose (Man), fucose (Fuc) and glucose (Glc) at a molar ratio of 3.5:1.4:1:1. In previous studies, FVPA1, FVPA2 and FVPB2 were obtained from *F. velutipes* using traditional extraction, separation and purification methods [8,10,27]. The three polysaccharides had different molecular weights and did not contain proteins. The monosaccharide composition of FVPA2 did not contain glucose.

Figure 2b shows that the FVPT1 had three major absorption peaks at 3416, 1651 and 1453 cm^−1^. The 3416 cm^−1^ peak indicates the polysaccharide’s hydroxyl stretching vibration. The strong absorption peak at 1615 cm^−1^ and the weak peak at 1453 cm^−1^ illustrate the characteristics of the polysaccharide. No uronic acids were present because of the absent peaks at 1730 cm^−1^ [30].

In Table 1 and Figure 2c,d, the GC-MS data indicate that the FVPT1 had a branched structure, including mannopyranose residue and glucopyranose residue, at the terminals; a main chain structure including 1,2-di-substituted fucose, 1,4,6-tri-substituted galactose, 1,3,6-tri-substituted galactose, 1,2-di-substituted fucose, 1,4-di-substituted galactose and 1,3-di-substituted glucose; and a fucose residue at the other terminal.

**Figure 3:**
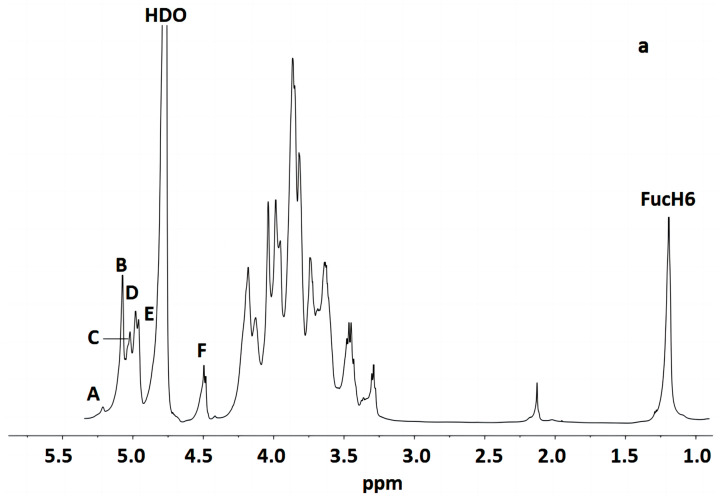

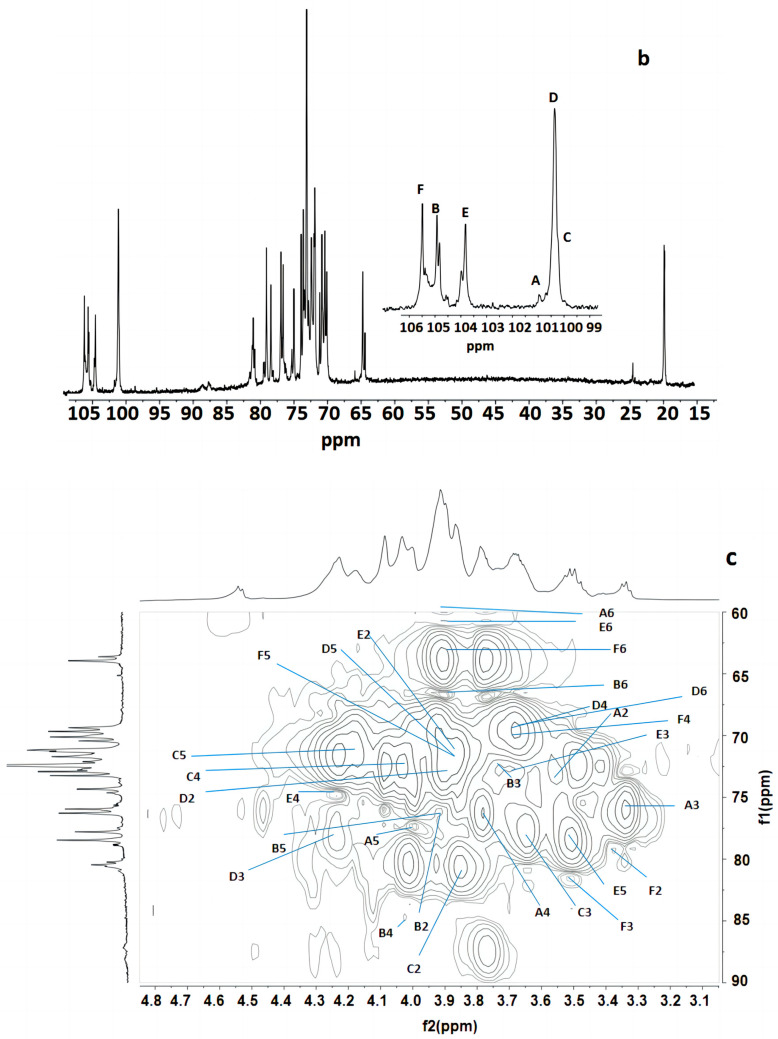
NMR of FVPT1 in deuterium oxide (D_2_O) at 27 °C. (**a**) The 600 MHz ^1^H NMR spectrum of FVPT1. The anomeric protons are labeled as (A)–(F). (**b**) The 150 MHz ^13^C NMR spectrum of the FVPT1 polysaccharide in D_2_O at 27 °C. (**c**) HMQC of the FVPT1 polysaccharide in D_2_O at 27 °C.

Figure 3 indicates that the polysaccharide structure of the FVPT1 included six sugar residues, named A–F, at δ 5.26 (single broad peak (br.s)), δ 5.12 (br.s), δ 5.07 (br.s), δ 5.03 (br.s), 5.01 (br.s) and δ 4.55 (double peaks (d), *J*_H-1, H-2_ = 6 Hz), respectively, corresponding to the signals at δ 101.55, 105.29, 100.73, 100.81, 104.20 and 105.87, respectively. A special signal at 1.24 (*J*_H-5, H-6_ = 6.42 Hz) showed the CH_3_-C group of the Fuc ^1^H signal, corresponding to the signals at C-6 of Fuc at δ 18.81.

The identities of monosaccharide residues A–F depended on the NMR, according to the chemical shifts and anomeric configurations of the six sugar residues in the FVPT1 structure.

Table 2 shows that the spin system for three sugar residues with the galacto configuration, A, B and D, was identified using the H-1/H-2 up to H-4 and H-6/H-5, H-4 correlations found in the ^1^H-^1^H COSY and TOCSY spectra. The downfield shifts of C-4 (δ 79.33) indicated that A was 1, 4- α-D-Gal*p*. C-4 (δ 84.70) and C-6 (δ 67.48) indicated that residue B was 1, 4, 6-α-D-Gal*p*. C-3 (δ 78.49) and C-6 (δ 69.32) indicated that residue D was (1, 3, 6)-α-D-Gal*p*.

Residue C has special signals at 1.24 (^1^H NMR) and δ 18.81 (^1^C NMR), indicating having α- fucose residue ether with δ 5.07, in addition to its small *J*_H-1, H-2_ value and GC-MS data. The C-2 (δ 87.43) carbon signals, compared with standard values, indicated that residue C was 1, 2-α-L-Fuc*p*.

Small values of *J*_H-1, H-2_ and *J*_H-2, H-3_, a large value of *J*_H-4, H-5_ and a small *J*_H-1, H-2_ value have identified that residue E was of D-mannosyl. That *J*_C-1, H-1_ = 170 Hz in HMBC indicated the presence of an α- Mannose configuration [31]. Except for C-1 (δ 104.20), no carbon signal was evident within the δ 76–88 range, indicating that E was a terminal α-D-mannopyranose [32].

The anomeric signal at δ 4.55 and the large value of *J*_H-1, H-2_ indicated that F was a β-linked residue. Proton chemical shifts from H-2 to H-6 were assigned from the COSY and HMQC spectra. Large values of *J*_H-2, H-3_ and *J*_H-3, H-4_ (9 Hz) and the typical H-1, H-2 and H-4 intracorrelations in the NOESY spectrum pointed out that residue F was D-glucopyranose [8]. C-3 (δ 81.66) carbon indicated that F was a 1, 3-link β-D Glc*p*.

**Figure 4:**
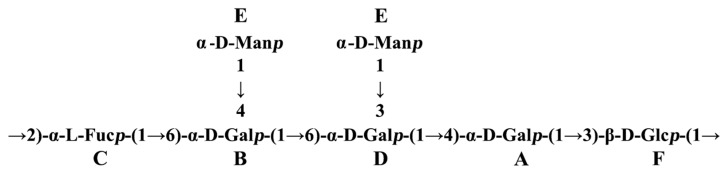
Repeating unit of FVPT1.

The sequence of the glycosyl residues was determined from the NOESY studies, followed by confirmation with HMBC experiments (Table 3 and Table 4). Based on the data presented above, the polysaccharide FVPT1 has the following repeating unit (Figure 4):

### 3.3. The Enhancement of FVPT1 to NO Release

**Figure 5:**
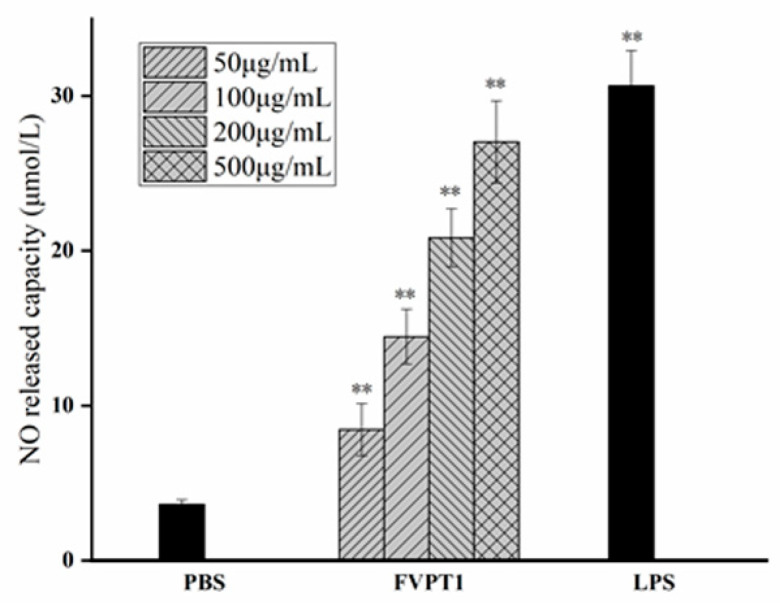
The enhancement of FVPT1 in NO release. The Raw264.7 cells were treated with FVPT1 (50, 100, 200 and 500 μg/mL) or 10 μg/mL of LPS for 48 h. ** *p* < 0.01 vs. the PBS group.

As a key signaling molecule and an important transduction factor in the immune system, NO’s content can be evaluated in the RNI levels of samples due to its relatively stable properties with RNI metabolites. As shown in Figure 5, FVPT1 could stimulate the level of NO in a dose-dependent manner at a certain concentration of 50–500 μg/mL compared to the negative control. Moreover, the 500 μg/mL FVPT1 showed obvious activation ability in NO production.

### 3.4. Effect on Cytokines Produced by Mouse Macrophages Activated by FVPT1

**Figure 6:**
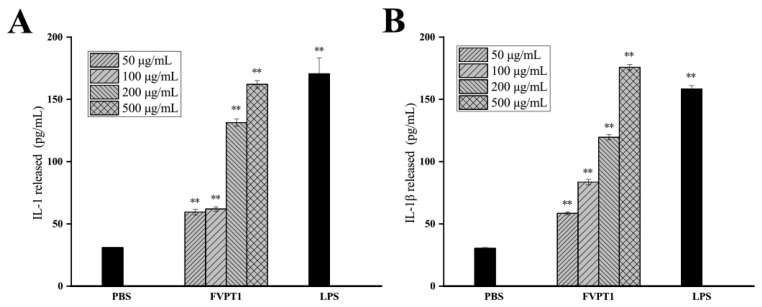
The promotion effects of FVPT1 on the cytokines released by RAW264.7. (**A**) Detection of IL-1 secretion with ELISA assay. (**B**) Detection of IL-1β with ELISA assay. ** *p* < 0.01 vs. the PBS group.

Activated macrophages can not only produce inflammatory mediators but also secrete cytokines such as IL-1 and IL-1β, which regulate local microenvironments and resist the invasion of adverse factors. An ELISA kit was used to detect the effect of FVPT1 on promoting macrophages to produce the aforementioned four cytokines, with the results showing that cocultures of FVPT1 could stimulate macrophages to secrete the IL-1β and IL-1 cytokines. Levels of IL-1β and IL-1 cytokines can be significantly promoted by a concentration of 500 μg/mL of FVPT1 (Figure 6). The results showed that FVPT1 could activate macrophages and secrete IL-1 and IL-1β cytokines in large quantities. In previous studies, FVPA1, FVPA2 and FVPB2 have been obtained from *F. velutipes* using traditional extraction, separation and purification methods [8,10,27]. Similarly to the in vitro immune efficacy of FVPT1, FVPA1 could stimulate Raw264.7 macrophages to secrete NO [10]. However, FVPA2 and FVPB2 could act on B cells or NK cells in vitro rather than on macrophages [8,27].

### 3.5. Lipid-Decreasing Effect of FVPT1 In Vivo

**Figure 7:**
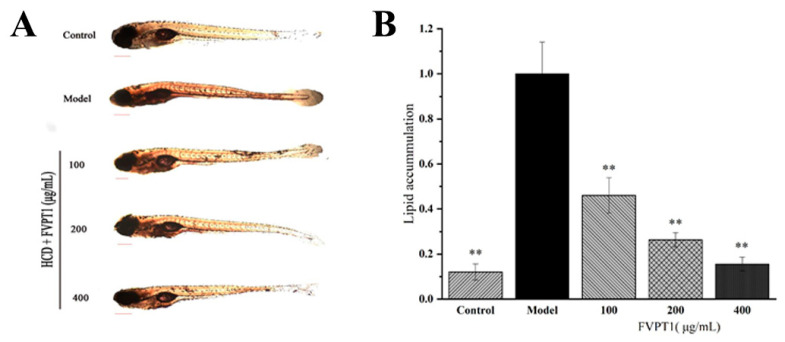
The lipid-decreasing effect of FVPT1 in vivo. (**A**) Representative photos of the whole-body stable lipid appearance of zebrafish larvae treated with FVPT1 at different concentrations (100, 200 and 400 μg/mL). (**B**) Lipid content was normalized to IOD data through blood vessels. ** *p* < 0.01 vs. the model group.

In recent years, zebrafish have often been used as models to clarify mechanisms of pharmacological action [33]. Previous studies have shown that the polysaccharides isolated from *Agaricus bisporus* and *Pleurotus eryngii* demonstrate significant blood lipid-lowering activity in zebrafish models [28,34]. Obviously, FVPT1 reduced the lipid content in the zebrafish larvae in our hyperlipidemia model as expected. The 100, 200 and 400 μg/mL FVPT1 lowered the lipid content to 53.89%, 73.76% and 83.89%, respectively, compared with 100% in the model group of zebrafish fed on yolk powder as a high-cholesterol diet (Figure 7). The results of the in vitro experiment suggested that FVPT1 has a good inhibitory effect on lipid accumulation.

## 4. Conclusions

In this study, FVPT1 with the molecular weight of ~1.64 × 10^4^ Da was separated using magnetic-field-assisted TPP and purified with S-300 (16 mm × 100 cm). Monosaccharide composition analysis showed that FVPT1 is composed of L-fucose, D-galactose, D-glucose and D-mannose at a molar ratio of 1.0:3.5:1.0:1.4. Methylation analysis and NMR spectroscopy established the polysaccharide repeating unit of FVPT1 and revealed that it was a new polysaccharide. Although several polysaccharide structures from *F. velutipes* have been reported recently, the polysaccharide moiety of FVPT1 represents a previously undocumented novel structure. In addition, the results of biological activity experiments have shown that FVPT1 exhibits good immunomodulatory activity by increasing nitric oxide, interleukin (IL)-1β and IL-1 secretion in macrophages and good hypolipidemic activity via appreciable lipid-lowering effects in a zebrafish larva hyperlipidemia model test. Research is ongoing in our laboratory to further characterize the nature of this polysaccharide and to investigate structure–activity relationships.

## Figures and Tables

**Table 1:** Methylation analysis data for FVPT1.

Methylated Sugar	Linkages	Molar Ratio	Major Mass Fragment (*m*/*z*)
2,3,6-M_3_-Gal*p*	1,4-linked-Gal*p*	0.87	43,71,87,99,102,113,118,129,142,173,203
2,3-M_2_-Gal*p*	1,4,6-linked-Gal*p*	1.01	43,59,74,89,99,118,127,142,159,201
3,4,6-M_3_-Fuc*p*	1,2-linked-Fuc*p*	1.08	43,59,72,87,101,118,130,143,190
2,4-M_2_-Gal*p*	1,3,6-linked-Gal*p*	0.76	87,117,129,189,233,305
2,3,4,6-M_4_-Man*p*	1-linked-Man*p*	1.71	43,71,87, 102,113,129,145,162,205
2,4,6-M_3_-Glc*p*	1,3-linked-Glc*p*	0.89	43,71,87, 101,117,129,145,157,133
2,3,4,6-M_4_-Gal*p*	1-linked-Gal*p*	0.13	43,59,71,87,102,113,129,145,205

**Table 2:** ^1^H and ^13^C NMR chemical shifts (ppm) of FVPT1 at 27 °C.

Residue	Proton or Carbon					
	H-1/C-1	H-2/C-2	H-3/C-3	H-4/C-4	H-5/C-5	H-6/C-6
A	5.26	3.56	3.78	3.35	4.00	3.76 ^a^, 3.90 ^b^
→4)-α-D-Gal*p*(1→	101.55	73.03	76.28	79.33	77.38	59.98
B	5.12	3.93	3.70	4.03	3.91	3.90 ^a^, 3.78 ^b^
→4,6)-α-D-Gal*p*(1→	105.29	76.18	72.77	84.70	76.25	67.48
C	5.07	3.88	3.65	4.02	4.18	1.24
→2)-α-L-Fucp-(1→	100.73	87.43	77.53	72.42	71.42	18.81
D	5.03	3.89	4.25	3.69	3.88	3.67 ^a^, 3.39 ^b^
→3,6)-α-D-Galp(1→	100.81	72.12	78.49	72.95	72.27	69.32
E	5.01	3.87	3.69	4.23	3.51	3.78 ^a^, 3.91 ^b^
α-D-Man*p*-(1→	104.20	71.57	72.77	74.57	78.33	61.02
F	4.55	3.38	3.51	3.67	3.87	3.77 ^a^, 3.90 ^b^
→3)-β-D-Glcp-(1→	105.87	79.41	81.66	69.79	71.67	63.95

^a^ Chemical shift for H-6a. ^b^ Chemical shift for H-6b.Values shown in bold font indicate linkage positions.

**Table 3:** Interglycosidic correlations from of FVPT1.

Residue	Proton	Proton Correlation ^a^
A: →4)-α-D-Gal*p*(1→	H-1 (δ 5.26)	3.51 (F:H-3), 3.78(A:H-3), 4.00(A:H-5)
	H-4 (δ 3.35)	5.03 (D:H-1)
B: →4,6)-α-D-Gal*p*(1→	H-1 (δ 5.12)	3.39 (D:H-6), 3.70 (B:H-3), 3.91 (B:H-5), 4.03 (B:H-4)
	H-4 (δ 4.03)	5.01 (E:H-1)
	H-6(δ 3.78)	5.07 (C:H-1)
C: 2)-α-L-Fuc*p*-(1→	H-1 (δ 5.07)	1.24 (C:H-6), 3.88 (C:H-2), 4.02 (C:H-4), 4.18 (C:H-5)
	H-2 (δ 3.88)	4.55 (F:H-1)
D: →3,6)-α-D-Gal*p*(1→	H-1 (δ 5.03)	3.35 (A:H-4), 3.39 (D:H-6), 3.69 (D:H-4), 3.89 (D:H-2), 4.25 (D:H-3)
	H-6 (δ 3.39)	5.12 (B:H-1)
	H-3 (δ 4.25)	5.01 (E:H-1)
E: α-D-Man*p*(1→	H-1 (δ 5.01)	3.69 (E:H-3), 3.87 (E:H-2), 4.03 (B:H-4), 4.25 (D:H-3)
F: →3)-β-D-Glcp-(1→	H-1 (δ 4.55)	3.51 (F:H-3), 3.67 (E:H-4), 3.88 (C:H-2)
	H-3 (δ 3.51)	5.26 (A:H-1)

^a^ Proton-correlation NOESY spectra are shown in bold font.

**Table 4:** Interglycosidic correlations of FVPT1.

Residue	Proton	Proton Correlation ^a^
A: →4)-α-D-Gal*p*(1→	H-1 (δ 5.26)	73.03 (A:C-2), 77.38 (A:C-5), 81.66(F:C-3)
	H-4 (δ 3.35)	100.81 (D:C-1)
B: →4,6)α-D-Gal*p*(1→	H-1 (δ 5.12)	67.48 (B:C-6), 69.32 (D:C-6), 72.77 (B,C-3)
	H-6 (δ 3.90)	100.73 (C:C-1)
C: →2)α -L-Fuc*p*-(1→	H-1 (δ 5.07)	67.48 (B:C-6), 71.42 (C:C-5)
	H-2 (δ 3.88)	105.87 (F:C-1)
D: →3,6)α-D-Gal*p*(1→	H-1 (δ 5.03)	69.32 (D:C-6), 72.12 (D:C-2), 78.49 (D:C-3),
	H-3 (δ 4.25)	104.20 (E:C-1)
E: α-D-Man*p*(1→	H-1 (δ 5.01)	74.57 (E:C-4), 78.49 (D:C-3)
F: →3)β-D-Glcp-(1→	H-1 (δ 4.55)	69.79 (F:C-4), 79.41 (F:C-2), 87.43 (C:C-2)
	H-3 (δ 3.51)	101.55 (A:C-1)

^a^ Proton-correlation HMBC spectra are shown in bold font.

## Data Availability

Data are contained within the article.
